# Dysphagia Aortica: A Rare Presentation of Infrarenal Abdominal Aortic Aneurysm in an Elderly Patient

**DOI:** 10.7759/cureus.71115

**Published:** 2024-10-08

**Authors:** Mahmoud Abughazal, Abdelsalam Dini, Mustafa Aljanabi, Mohammed Al-Banna, Moustafa Abouelkheir

**Affiliations:** 1 Emergency Medicine, United Lincolnshire Hospitals NHS Trust, Boston, GBR; 2 Emergency Department, Pilgrim Hospital, Boston, GBR

**Keywords:** acute aortic syndromes, atypical presentation of aortic dissection, dysphagia aortica, endovascular intervention, infrarenal abdominal aorta, leaking aortic abdominal aneurysm

## Abstract

This article explores the challenges in diagnosing dysphagia aortica through the case of an 89-year-old man with a medical history of hypertension, ischemic heart disease, and chronic kidney disease. Over a three-month period, the patient experienced progressively worsening dysphagia and indigestion. On the day of presentation, his condition further deteriorated, marked by hypotension. Point-of-care ultrasound (POCUS) identified a large infrarenal abdominal aortic aneurysm (AAA) measuring 13 cm, which was later confirmed by CT to involve dissection and a contained rupture. This case highlights the rare presentation of dysphagia secondary to an infrarenal AAA, known as dysphagia aortica, emphasizing the importance of considering AAAs in elderly patients with vague gastrointestinal symptoms. Despite prompt diagnosis and referral for vascular surgery, the patient's prognosis was poor due to advanced age, frailty, and multiple comorbidities. Unfortunately, he passed away a few hours after presenting to the emergency department. This article underscores the critical role of imaging modalities such as POCUS and CT in identifying life-threatening conditions like AAAs, and it advocates for a comprehensive differential diagnosis in older adults while also serving as a reminder of the atypical presentations of AAAs.

## Introduction

This case exemplifies an unusual presentation of an infrarenal abdominal aortic aneurysm (AAA) in an elderly male with multiple significant comorbidities, including hypertension, ischemic heart disease, and stage 3 chronic kidney disease. Generally, AAAs result from a degenerative process associated with aging, predominantly affecting males with risk factors such as tobacco use, Caucasian ethnicity, family history, and peripheral artery disease [1]. In most cases, these aneurysms are asymptomatic until they rupture, which typically results in acute abdominal or back pain accompanied by hemodynamic instability [2]. However, the patient in this scenario exhibited more subtle and gradual symptoms over three months, such as worsening dysphagia, indigestion, and a general decline in health. This condition is categorized as dysphagia aortica, an obstruction to swallowing caused by the external compression of the esophagus by the aorta itself, often due to an enlarged or tortuous aorta [3].

Such atypical manifestations complicate the timely identification of AAA and underscore the need for heightened clinical vigilance when encountering nonspecific gastrointestinal or respiratory symptoms in older patients. The patient's reluctance to seek medical help, combined with the absence of classic AAA symptoms, likely contributed to the delay in diagnosis. In this case, dysphagia may have resulted from pressure exerted by the expanding aneurysm on nearby structures, such as the esophagus [4]. Although not frequently reported, dysphagia has been documented in rare instances where aneurysms compress mediastinal or retroperitoneal elements [5].

Upon initial evaluation, the patient appeared stable with normal oxygen saturation levels and heart rate; however, hypotension and hypothermia raised concerns about potential shock despite initially stable vital signs. Point-of-care ultrasound (POCUS) was crucial in promptly identifying a large infrarenal aneurysm measuring 13 cm in diameter. POCUS has increasingly proven its value in emergency settings for rapidly diagnosing life-threatening conditions such as AAA, where time is critical [6]. The diagnosis of an infrarenal AAA with dissection and hemorrhage was subsequently confirmed through a CT scan, the gold standard for confirming AAA diagnoses and assessing their severity [7]. CT findings revealed significant periaortic hemorrhagic fluid, consistent with a contained rupture. In this scenario, hemorrhaging was temporarily contained by surrounding tissues, leading to more subtle clinical manifestations, as observed in this patient [8]. However, despite initial containment measures, there remained a substantial risk of further rupture and rapid clinical deterioration [9].

## Case presentation

An 89-year-old male with a medical history of essential hypertension, ischemic heart disease, and stage 3 chronic kidney disease presented to the emergency department after a three-month history of progressively worsening gastrointestinal symptoms. He reported a three-month history of dysphagia, primarily for solid foods rather than liquids, and persistent indigestion, which did not improve despite frequent use of antacid syrup. On the day of presentation, he experienced abdominal discomfort, distention, and a left-sided abdominal swelling, along with an acute decline in his overall health. The patient had been reluctant to seek medical care, avoiding hospital visits and consultations with his general practitioner.

On initial examination, the patient's vital signs were relatively stable, with a pulse of 71 beats per minute, a respiratory rate of 18 breaths per minute, a blood pressure of 97/65 mmHg with a mean arterial pressure of 76, and an oxygen saturation of 100%. However, signs of hypotension and hypothermia raised concerns for impending shock. Blood samples were collected, and intravenous fluids were administered, leading to mild improvement. Despite this, his condition rapidly worsened, requiring transfer to the resuscitation area approximately two hours after presentation.

**Figure 1:**
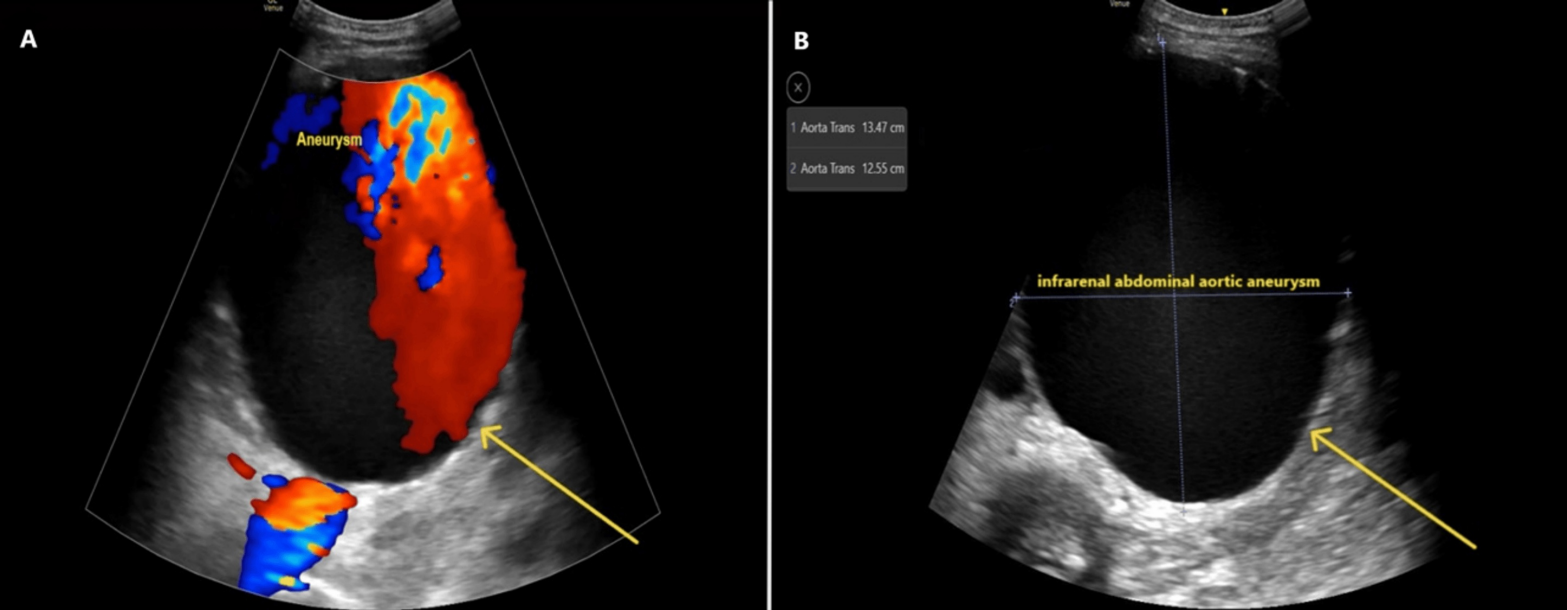
Bedside Doppler abdominal ultrasound demonstrated a 13.47 × 12.55 cm abdominal aortic aneurysm in anteroposterior and transverse dimensions, respectively, as denoted by the yellow arrows. (A) Colour Doppler ultrasound. (B) Doppler ultrasound (without colour)

**Figure 2:**
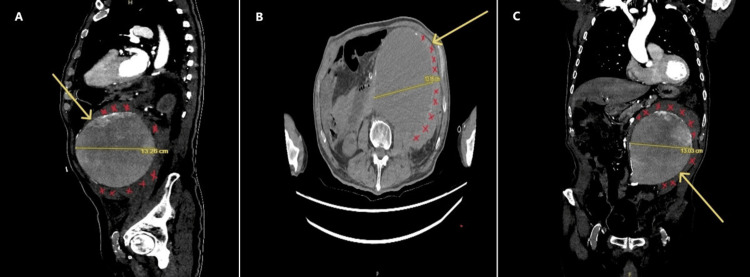
Contrast-enhanced CT of the abdomen and pelvis in sagittal view (A), axial view (B), and coronal view (C) demonstrates a large infrarenal abdominal aortic aneurysm, with maximum axial dimensions of 13.16 × 13.26 cm (transverse × anteroposterior), extending approximately 14.1 cm in length, as denoted by the yellow arrows. The periaortic hemorrhagic fluid and surrounding fat stranding are marked with red "X"s.

Laboratory results, as shown in Table 1, revealed a drop in hemoglobin levels from 11.9 g/dL (recorded a year ago) to 8.6 g/dL, along with acute kidney injury. Meanwhile, C-reactive protein (CRP), white blood cell (WBC) count, and electrolyte levels remained within normal limits, raising concerns about a possible subtle hemorrhage. POCUS revealed a large infrarenal AAA measuring 13 cm in diameter (highlighted in Figure 1). A subsequent CT scan confirmed the presence of an infrarenal AAA with dissection, accompanied by significant periaortic hemorrhagic fluid and fat stranding, as illustrated in Figure 2.

**Table 1:** Lab parameters. Hb: hemoglobin; WBC: white blood cell; Cr: creatinine; Na: sodium; K: potassium; CRP: C-reactive protein; GFR: glomerular filtration rate

Lab parameters	Value	Normal range
WBC count	12.7	4.5-11 × 10^9^/L
Platelets	180	150-400 × 10^3^/µL
Urea	14.3	2.1-8.5 mmol/L
Cr	213	59-104 μmol/L
Na	132	136-145 mmol/L
Hb	8.6	14-18 g/L
K	5.2	3.6-5.2 mmol/L
CRP	2.9	Below 5 mg/L
GFR	23	90-100 ml/min

The patient was urgently referred to the vascular surgery team for comprehensive assessment and further management. However, after a critical evaluation of the risks versus benefits of surgery and endovascular intervention for this elderly patient, particularly given his significant comorbidities, it was determined that he was not a candidate for surgical or endovascular intervention. This decision was influenced by the combination of the patient's advanced age, acute deterioration, and expressed end-of-life preferences.

Despite prompt referral and initial management in the emergency department, including fluid resuscitation, his condition continued to deteriorate. Ultimately, he succumbed to complications related to the large infrarenal AAA with dissection and associated hemorrhage.

In this case, the patient's condition progressively worsened. His pre-existing Do Not Resuscitate (DNR) order, along with his Recommended Summary Plan for Emergency Care and Treatment (ReSPECT) form, underscored the importance of aligning medical interventions with his end-of-life care preferences. The patient passed away shortly thereafter.

## Discussion

Typically, AAAs develop through a degenerative process associated with aging, particularly in males with vascular risk factors. Most AAAs remain asymptomatic until rupture, which classically manifests as acute abdominal or back pain and hemodynamic instability [5]. However, this patient presented with more subtle and insidious symptoms, including progressive dysphagia, indigestion, and a general decline in health over three months. This presentation falls under the term dysphagia aortica, which describes an obstruction in swallowing due to external aortic compression, commonly caused by a dilated, tortuous, or aneurysmal aorta [10]. Such atypical presentations complicate the timely diagnosis of AAA and highlight the need for heightened clinical suspicion in patients with vague gastrointestinal or respiratory symptoms, particularly in the elderly population.

The patient's reluctance to seek medical attention, combined with the absence of typical AAA symptoms, likely contributed to the delayed diagnosis. Dysphagia, as noted in this case, could have been caused by a mass effect from the expanding aneurysm, exerting pressure on adjacent structures such as the esophagus [5]. While dysphagia is not a commonly reported symptom of AAA, it has been described in rare cases where aneurysms exert pressure on mediastinal or retroperitoneal structures [2].

On initial assessment, the patient appeared stable, with normal oxygen saturation and a heart rate within the normal range. However, hypotension and hypothermia, despite initially stable vitals, raised concerns of impending shock. The use of POCUS was critical in quickly identifying the large infrarenal aneurysm, which measured 13 cm in diameter. POCUS has become an increasingly valuable tool in emergency settings for the rapid diagnosis of life-threatening conditions like AAA, especially when time is of the essence [11].

The diagnosis of an infrarenal AAA with dissection and hemorrhage was further confirmed by a CT scan, which remains the gold standard for diagnosing AAA and assessing its severity [12]. The CT findings, which revealed significant periaortic hemorrhagic fluid, were indicative of a contained rupture. This type of rupture, where the hemorrhage is temporarily confined by surrounding tissues, can present with more subtle clinical signs, as observed in this patient [12]. However, despite initial containment, the risk of further rupture and rapid deterioration remained high [13].

The patient's chronic kidney disease likely exacerbated his clinical picture. Patients with chronic kidney disease are at higher risk for poor outcomes in AAA cases due to impaired renal function, which complicates both medical management and surgical intervention [14].

In this case, despite timely referral to the vascular surgery team and the initiation of intravenous fluids, the patient continued to deteriorate. His pre-existing DNR order, based on discussions regarding his poor prognosis and limited potential for recovery, guided the decision to focus on comfort measures. The ReSPECT form emphasized aligning interventions with the patient's end-of-life preferences, although it was noted that DNR orders can always be reassessed if the situation changes.

This case serves as a reminder that while large AAAs typically present with acute pain and shock, they can also present atypically, as seen in this patient with dysphagia and delayed symptom progression. The use of bedside imaging modalities such as POCUS and the availability of CT scanning are essential in diagnosing life-threatening conditions. However, even with optimal diagnostic tools and interventions, the prognosis for ruptured or dissecting AAAs, particularly in elderly and frail patients, remains poor [15].

## Conclusions

This case report highlights the unusual presentation of an infrarenal AAA with dysphagia aortica in an elderly male patient. The key findings of this case include the gradual onset of nonspecific gastrointestinal symptoms such as dysphagia and indigestion, which delayed the diagnosis of a large, life-threatening aneurysm. The use of POCUS and CT imaging was crucial in promptly identifying the aneurysm and its complications. However, the patient's advanced age, frailty, and comorbidities significantly impacted his prognosis. This case underscores the need for clinicians to consider AAAs in the differential diagnosis for older adults with vague gastrointestinal symptoms, as early recognition is vital for timely intervention and improving patient outcomes.

## References

[1] (2024). Abdominal aortic aneurysm. https://www.mayoclinic.org/diseases-conditions/abdominal-aortic-aneurysm/symptoms-causes/syc-20350688.

[2] Sakalihasan N, Limet R, Defawe OD (2005). Abdominal aortic aneurysm. Lancet.

[3] Johnston KW, Rutherford RB, Tilson MD, Shah DM, Hollier L, Stanley JC (1991). Suggested standards for reporting on arterial aneurysms. Subcommittee on Reporting Standards for Arterial Aneurysms, Ad Hoc Committee on Reporting Standards, Society for Vascular Surgery and North American Chapter, International Society for Cardiovascular Surgery. J Vasc Surg.

[4] Kim JH, Jang SW, Kim DB (2009). A patient with dysphagia due to an aortic aneurysm. Korean Circ J.

[5] Grimaldi S, Milito P, Lovece A, Asti E, Secchi F, Bonavina L (2022). Dysphagia aortica. Eur Surg.

[6] Suleman M, Pyuza J, Sadiq A, Lodhia J (2022). Aortic aneurysm: an uncommon cause of dysphagia. SAGE Open Med Case Rep.

[7] Abdul Haziz SR, Bickle I, Chong VH (2015). Dysphagia aortica: a rare cause of dysphagia. BMJ Case Rep.

[8] Tayal VS, Graf CD, Gibbs MA (2003). Prospective study of accuracy and outcome of emergency ultrasound for abdominal aortic aneurysm over two years. Acad Emerg Med.

[9] Lederle FA, Johnson GR, Wilson SE (2000). The aneurysm detection and management study screening program: validation cohort and final results. Aneurysm Detection and Management Veterans Affairs Cooperative Study Investigators. Arch Intern Med.

[10] Taofan T, Dakota I, Adiarto S (2024). Case report: emergency endovascular management of a ruptured giant abdominal aortic aneurysm with severely angulated and conical shaped neck using novel multiple stiff wire technique. F1000Research.

[11] Moxon JV, Parr A, Emeto TI, Walker P, Norman PE, Golledge J (2010). Diagnosis and monitoring of abdominal aortic aneurysm: current status and future prospects. Curr Probl Cardiol.

[12] Skandhan A, Niknejad M, Bos D (2024). Abdominal aortic aneurysm rupture. https://doi.org/10.53347/rID-25600.

[13] Kuivaniemi H, Tromp G, Prockop DJ (1991). Genetic causes of aortic aneurysms. Unlearning at least part of what the textbooks say. J Clin Invest.

[14] Sarafidis P, Martens S, Saratzis A (2022). Diseases of the aorta and kidney disease: conclusions from a kidney disease: improving global outcomes (KDIGO) controversies conference. Cardiovasc Res.

[15] Alasasfeh I, Abudawood R, Hwidi B, Al-Shami R (2024). Ruptured abdominal aortic aneurysm discovered by pocket-sized ultrasound in a low resource setting: a case report. Int J Emerg Med.

